# Role of diffusion tensor imaging in analyzing the neural connectivity of the parieto-insular vestibular cortex in pusher syndrome: As case report

**DOI:** 10.1097/MD.0000000000019835

**Published:** 2020-04-17

**Authors:** Sang Seok Yeo, Sung Ho Jang, Seunghue Oh, Jung Won Kwon

**Affiliations:** a Department of Physical Therapy, College of Health Sciences, Dankook University; b Department of Physical Medicine and Rehabilitation, College of Medicine, Yeungnam University; c Department of Health, Graduate School, Dankook University, Republic of Kore.

**Keywords:** central vestibular disorder, diffusion tensor imaging, parieto-insular vestibular cortex, pusher syndrome, vestibular pathway

## Abstract

**Rationale:**

Pusher syndrome is a disorder of postural control. It is associated with unilateral lesions on central vestibular system. In the current study, we attempted to identify and investigate neural connectivity of the parieto-insular vestibular cortex in a patient with pusher syndrome, using diffusion tensor imaging.

**Patient concerns:**

A 60-year-old male patient had left hemiplegia due to an infarction on right premotor cortex, primary motor cortex, corona radiata and temporal and occipital lobe. The patient had severe motor weakness in left upper and lower limb, left side neglect and significant pusher syndrome.

**Diagnosis:**

Patient was diagnosed with left hemiplegia due to an infarction in the right middle cerebral artery territory at the neurology department of a university hospital.

**Interventions:**

One patient and 5 control subjects of similar age participated. Diffusion tensor imaging data were acquired at 4-month and 12-month after the initial injury.

**Outcomes:**

Fractional anisotropy, mean diffusivity, and tract volume (TV) were measured. TV values in both affected and unaffected hemispheres of the patient were significantly decreased at 4-month compared to those of control subjects. In the unaffected hemisphere of the patient, TV value showed significant increase at 12-month compared to that at 4-month. Although the TV value at 12-month of the affected hemisphere was out of reference range, TV was considerably increased compared to that at 4-month. Mean values for fractional anisotropy or mean diffusivity in 2 hemispheres did not show significant difference compared to those of control subjects regardless of month.

**Lessons:**

Restoration of an injured projection pathway between the vestibular nuclei and parieto-insular vestibular cortex with recovery of pusher syndrome was found in a patient with stroke.

## 1 Introduction

Stroke patients are frequently impaired in balance and postural control. They fail to maintain postural stability of the trunk or their gravitational line moves toward the nonparetic side as a result of sensory loss, perceptual dysfunction, deficit of internal processes of sensory integration and postural control.^[1–4]^ However, some patients may show a specific postural disorder. They use the nonparetic arm or leg to actively push from nonparalyzed toward the paralyzed side and resist any attempts to passively correct their tilted body posture (also termed “Pusher Syndrome”).^[1,5,6]^ Patients with pusher syndrome have several problems in the performance of independent and functional activities of daily living.^[7–9]^ In addition, they need more time to recover their motor ability, thus prolonging inpatient rehabilitation.^[4,10,11]^

Many imaging studies by magnetic resonance imaging or computed tomography have reported that posterior-lateral thalamus, internal capsule, supplementary motor area, upper parietal lobe, globus pallidus, and parieto-insular vestibular cortex (PIVC) are relevant structures to distinct pushing behavior.^[12–14]^ The PIVC is a main region of the central vestibular system, including the insular and retroinsular cortex, Sylvian fissure, frontoparietal operculum, and the superior temporal gyrus.^[15–17]^ Patients with lesions in this area show impairment of spatial orientation, spatial attention, and balance control.^[18]^ They depend on integration of multisensory (that is, visual, vestibular, and somatosensory) input. Similarly, pusher syndrome is typically associated with unilateral lesions on the posterior nuclei of thalamus, posterior insular cortex, superior temporal gyrus, postcentral gyrus, and inferior parietal lobule.^[14,19,20]^ Theses lesion sites contain components of the multisensory cortical network as central vestibular system.^[21]^ However, the role of the central vestibular system in posture control of pusher syndrome is not properly clarified yet.

Recently, several studies of vestibular connectivity using diffusion tensor imaging (DTI) have found a congruent functional and structural link between the vestibular nuclei and the PIVC.^[22–25]^ Vestibular pathways travel from the vestibular nuclei into the vestibule cerebellum, brainstem, thalamus, and vestibular cortex areas. Some pathways travel via dorsolateral thalamus to the posterior part of the insula.^[16,26,27]^ Connected areas are the lesion sites that cause the pusher syndrome.^[5,28–31]^ However, the projection vestibular pathway from the vestibular nuclei via thalamus to the PIVC in patients with pusher syndrome has not been reported yet. In the current study, we attempted to identify and investigate neural connectivity of the PIVC in a patient with pusher syndrome using DTI.

## 2 Case presentation

**Figure 1:**
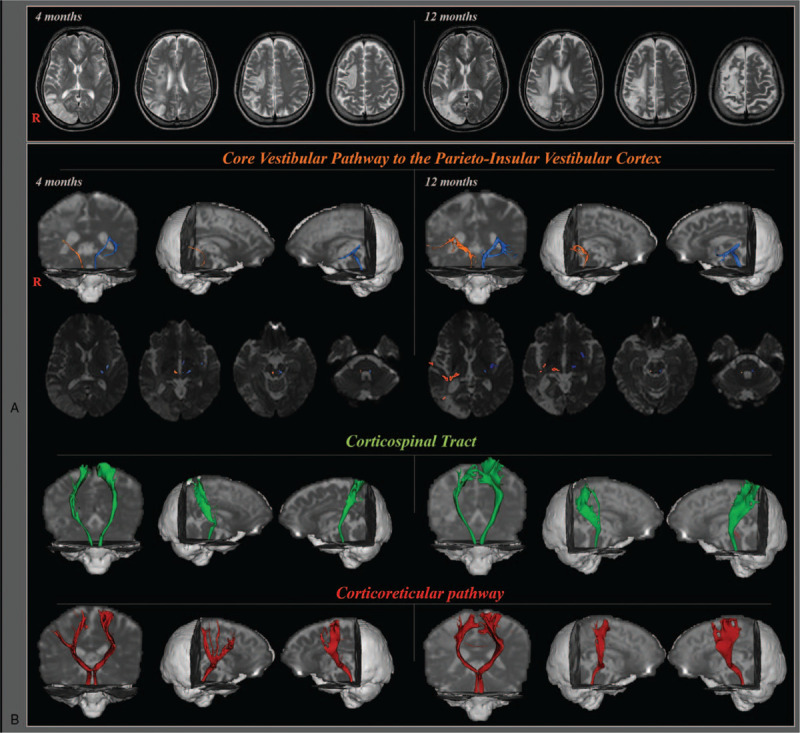
(A) T2-Weight image of a patient at 4 months and 12 months after onset shows infarction on the right premotor cortex, primary motor cortex, corona radiata, and temporal and occipital lobe. (B) Results of diffusion tensor tractography of core vestibular pathway to the parieto-insular vestibular cortex, corticospinal tract and corticoreticuar pathway in the patient a 4 months and 12 months after onset.

A 60-year-old male patient was diagnosed with left hemiplegia due to an infarction in the right middle cerebral artery territory at the neurology department of a university hospital. T2-weighted MR images showed lesions in the right premotor cortex, primary motor cortex, corona radiata, and temporal and occipital lobe at 4 months after onset (Fig. 1A). MR images taken at 12 months after injury showed greater extent of lesion due to Wallerian degeneration or encephalomalatic lesion (Fig. 1A).^[32]^ Four months following the infarction, he was transferred to the rehabilitation department of the same university hospital with severe motor weakness in the left upper and lower limb (functional ambulation category: 0 grade, total motricity index: 28 score) with left side neglect and significant pusher syndrome (scale for contraversive pushing: sitting-posture: 0.25, standing-posture: 0.75, standing-resistance: 1).^[33–35]^ Five age-matched control subjects (2 males and 3 females with mean age of 58.8 years, age range: 54–63 years) without history of neurologic disease were recruited for the control group. The patient and control subjects provided signed, informed consent for publication of the case. The study protocol was approved by the Institutional Review Board of our university hospital (approval number: YUH-12–0421-O60).

### 2.1 Diffusion tensor image

DTI data was acquired at 4-month and 12-month after the initial injury using a 6-channel head coil on a 1.5 T Philips Gyro scan Intera (Philips, Best, The Netherlands) with single-shot echo-planar imaging. For each of the 32 non-collinear diffusion sensitizing gradients, 67 contiguous slices were acquired parallel to the anterior commissure-posterior commissure line. Imaging parameters were as follows: acquisition matrix = 96 × 96; reconstructed matrix = 192 × 192; field of view = 240 × 240 mm^2^; TR = 10,726 ms; TE = 76 ms; parallel imaging reduction factor (SENSE factor) = 2; EPI factor = 49; b = 1000 s/mm^2^; NEX = 1; and a slice thickness of 2.5 mm without gap (acquired voxel size 1.3 × 1.3 × 2.5 mm^3^).

### 2.2 Probabilistic fiber tracking

Diffusion-weighted imaging data were analyzed using the Oxford Centre for Functional Magnetic Resonance Imaging of the Brain (FMRIB) Software Library (FSL; www.fmrib.ox.ac.uk/fsl). Affine multi-scale 2-dimensional registration was used for correction of head motion effect and image distortion due to eddy current. Fiber tracking was performed using a probabilistic tractography method based on a multifiber model and applied in the present study utilizing tractography routines implemented in FMRIB Diffusion (5000 streamline samples, 0.5 mm step lengths, curvature thresholds = 0.2).

The projection vestibular pathway to the PIVC was determined by selecting fibers passing through the seed region and 2 target regions of interest (ROIs). To reconstruct the projection vestibular pathway to the PIVC, we placed the seed ROI on the vestibular nuclei at the level of the pons corresponding to Schwalbe's nucleus and Deiters’ nucleus and the target ROI on the PIVC based on a previous study.^[22,24,25]^ Each descending motor pathway was determined by selecting fibers passing through seed and target ROI as follows: corticospinal tract (CST), seed ROI- CST portion of the pontomedullary junction on color map, target ROI 1 - CST portion of the anterior mid-pons, target ROI 2 – primary motor cortex^[36]^; corticoreticular pathway (CRP), seed ROI- reticular formation of the medulla, target ROI 1 - the midbrain tegmentum, target ROI 2 - premotor cortex.^[37]^

There were 5000 samples generated from the seed voxel. Results were visualized at the threshold of 1 streamline through each voxel for analysis. Fractional anisotropy (FA), mean diffusivity (MD), and tract volume (TV) of the projection vestibular pathway to the PIVC were measured. DTI parameters showing a deviation of more than 2 standard deviations (SD) of that of normal control values were defined as abnormal.

## 3 Results

Scale for contraversive pushing score and motor function in the patient are shown in Table 1. In sitting position, the score of posture showed decrease at 12-month compared to that at 4-month. In contrast, scores of extension and resistance were both 0 point, which was not different between 4-month and 12-month. In standing position, scores of posture and resistance were significantly decreased from 4-month to 12-month. However, there was no significant difference in the score of extension between 4-month and 12-month. As a result of motor function analysis, functional ambulation category was significantly increased at 12-month compared to that at 4-month. Total motricity index that examined 6 movements showed significant increase at 12-month compared to that at 4-month.

**Table 1:**
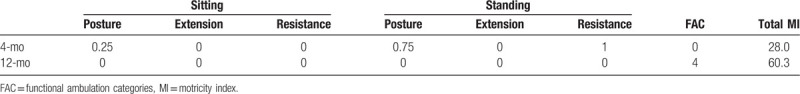
Changes of scale for contraversive pushing score and motor function in a patient (Unit: score).

On diffusion tensor tractography (DTT) of a patient, the projection pathway between the vestibular nucleus and the PIVC showed changes in Figure 1B. Results of DTI analysis showed that TV values in both affected and unaffected hemispheres of the patient were significantly decreased at 4-month compared to those of control subjects. In the unaffected hemisphere of the patient, TV value showed significant increase at 12-month compared to that at 4-month. Therefore, TV value of the unaffected hemisphere at 12-month was within the reference range. It was not significantly different from that of control subjects. Although TV value at 12-month of the affected hemisphere was out of reference range, the result of TV was considerably increased compared to that at 4-month. Mean values for FA or MD in the 2 hemispheres did not show significant difference compared to those of the control subjects regardless of month (Table 2).

**Table 2:**
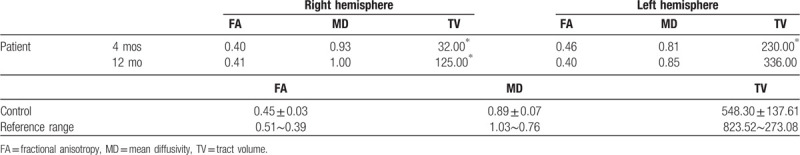
Comparison of diffusion tensor imaging parameters in a patient and control subjects.

## 4 Discussion

In this study, DTT changes in configuration and parameters of the projection pathway between the vestibular nucleus and the PIVC were tracked in a patient who showed marked recovery of pusher syndrome and motor function (Fig. 1B). In terms of DTT parameters, FA or MD values of the vestibular pathway to the PIVC were not significantly different from those of the control group. In 4-month DTT, TV values showed significant decrement in affected and unaffected hemisphere compared to those of the normal control group. On the other hand, in the 12-month DTT, TV value of the unaffected hemisphere was increased to within normal range while TV value of the affected hemisphere was not in the normal range, but it increased nearly 4 times compared to that at 4-month DTT. The FA value acts as a proxy measure of white matter organization by indicating the degree of directionality of water diffusion. The MD value indicates the magnitude of water diffusion in tissue which can increase with some forms of pathology or neuronal injury. Tract volume is determined by the number of voxels included in a reconstructed neural pathway by DTT. Therefore, decreased TV compared with normal range indicates injury of a neural tract whereas TV increase indicates recovery of an injured neural tract. Results of this study suggest that the pusher syndrome that lasted for up to 4 months after the onset of stroke is probably due to injury of the projection pathway between the vestibular nucleus and the PIVC in both hemispheres. Restoration of the pusher syndrome could be related to restoration of the normal range of the projection pathway of the unaffected hemisphere and considerable increment of TV value in the affected hemisphere.

Many previous studies have reported that head and body posture and balance are impaired following an acute unilateral stroke which appears to actively push out the non-paralyzed side to maintain an upright posture.^[1,2,5,6,38]^ However, there are few studies on whether pusher syndrome after stroke is related to vestibular dysfunction. In 2006, Johannsen et al reported that acute stroke patients with pusher syndrome due to cortical and subcortical lesion preserved the thalamus.^[14]^ According to results of their study, the insular cortex and part of the postcentral gyrus were areas of overlapping lesion in patients with pusher syndrome. The insular cortex and postcentral gyrus are known to have important reciprocal connection in human vestibular system.^[20]^ Especially, the PIVC as part of insular cortex is a brain area with prominent vestibular inputs. It is involved in processing of visual motion, particularly motion coherent with gravitational vector. In 2012, Baier et al reported that the anatomical cortical region could induce the pusher syndrome. They also reported the association of tilt of subjected visual vertical (SVV) and pusher syndrome in patients with acute stroke.^[12]^ They suggested that 42% of patients with right hemispheric stroke had pusher syndrome and 25% of them had left hemispheric stroke. Additionally, patients with acute stroke with pusher syndrome showed an increase in SVV tilt toward the contralesional side. In addition, patients with right side lesion showed increased left side tilt of SVV while patients with left side lesions showed opposite patterns. Results of these previous studies are consistent with results of current study, showing significant pusher syndrome following injury of vestibular projection pathway and recovery of pusher syndrome with restoration of vestibular projection pathway. However, several limitations of this study should be considered. First, because this is a case report, results of this study are descriptive in nature. Second, DTI may underestimate fiber tracts. Regions of fiber complexity and crossing can prevent full reflection of the underlying fiber architecture by DTI. Third, we could not precisely set the location of ROIs because of cramped size of vestibular nuclei.

In conclusion, restoration of an injured projection pathway between the vestibular nuclei and PIVC with recovery of pusher syndrome was demonstrated in a patient with stroke. We believe that this study will help clinical management and research for patients with pusher syndrome. Further studies involving larger case numbers and more detailed clinical correlation with injury of the vestibular projection pathway are needed.

## Author contributions

Sang Seok Yeo: manuscript development, data acquisition and manuscript writing.

Sung Ho Jang: conceiving and designing the study, manuscript development.

Seung Hue Oh: data acquisition and data analysis, manuscript writing

Jung Won Kwon: manuscript development, funding, manuscript writing and manuscript authorization

**Conceptualization:** Sung Ho Jang.

**Data curation:** Sang Seok Yeo, Seung Hue Oh.

**Formal analysis:** Seung Hue Oh.

**Funding acquisition:** Jung Won Kwon.

**Methodology:** Sung Ho Jang.

**Writing – original draft:** Sang Seok Yeo, Jung Won Kwon.

**Writing – review & editing:** Sung Ho Jang, Jung Won Kwon.
